# Nanomaterials for Potential Uses in Extraterrestrial Environments

**DOI:** 10.3390/nano14100893

**Published:** 2024-05-20

**Authors:** Angelo Nicosia, Placido Mineo

**Affiliations:** 1 Department of Chemical Sciences and INSTM UdR of Catania, University of Catania, V.le A. Doria 6, I-95125 Catania, Italy; angelo.nicosia@unict.it; 2 Institute for Chemical and Physical Processes, National Research Council (IPCF-CNR), Viale F. Stagno d’Alcontres 37, I-98158 Messina, Italy; 3 Institute of Polymers, Composites and Biomaterials, National Research Council (IPCB-CNR), Via P. Gaifami 18, I-95126 Catania, Italy

Over the past decades, the development of nanomaterials has played an important role in the most intriguing aspects of new technologies in several scientific fields, such as nanoelectronics, nanomedicine [1,2,3,4], environmental remediation [5,6,7], and sensing [8,9,10], just to mention a few. Among the future challenges, perhaps the most difficult ones concern space exploration activities and the use of ordinary materials in extraterrestrial environments. Indeed, the main space agencies are working on a series of cutting-edge projects with the colonization of the Moon and Mars as their final goal [11].

In this context, it might not be possible to use common materials due to the chemical and physical stresses caused by extreme environmental conditions, such as low gravity, solar wind, and cosmic rays (ionizing radiation and high-energy ions) [12,13,14], that can deeply and negatively impact the structural and chemico-physical properties of the used materials [15]. Therefore, understanding the chemico-physical effects of extreme environmental conditions on materials is critical in terms of developing advanced solutions suitable for the different aspects and challenges of space missions.

Polymer-based materials play a key role in the development of infrastructures designed for extraterrestrial environments due to their ease of processability and low weight. As an example, different polymers and composites are interesting to use as extraterrestrial construction materials because they can be produced in situ by exploiting the local resources of the extraterrestrial environments [16]. Nevertheless, their durability under harsh conditions (e.g., thermal cycles and vacuum, as well as space stresses such as solar wind, cosmic rays, and UV radiation) must be improved [17].

For instance, as shown by Mezzina et al. [18], mimicked solar wind bombardment produces significant structural changes on polymethylmethacrylate (PMMA) that affect the properties and chemical structure of its backbone, determining a degradation route, in turn leading to the formation of a brittle crosslinked polymer.

Considering the issues of the extraterrestrial environment, radiation shielding is also crucial in order to ensure the liveability of spacecraft cockpits and human habitats. Space agencies are aware of the risks of ionizing radiation exposures for their crews, inducing deleterious late effects on human organisms [19]. These might be even worse, considering long-term expeditions. Different materials have been investigated in the context of passive radiation shielding. As an example, NASA has used composite panels of aluminum and polyethylene to shield the crew on the International Space Station [20,21]. By exploiting the radiation tolerance of the carbon-based systems, the loads of suitable fillers within the polymer matrix might improve the performance of the materials. With regard to this, Zaccardi et al. [22] investigated the improvements in the radiation shielding properties of a medium-density polyethylene when loaded with graphene nanoplatelets and/or multi-walled carbon nanotubes, showing the efficiency of the proposed nanocomposites that undergo negligible changes in their chemico-physical properties after proton bombardment.

The presence of several destructive agents is not only a problem of the extreme outer space environment that future space missions must face. For example, satellites for communications, meteorology, observations, etc. are located in the low earth orbit (LEO), where the density of residual oxygen is sufficient to induce chemical and physical material erosion. In fact, due to the UVC-triggered photodissociation of atmospheric oxygen in LEO, the most abundant gaseous species in this region is represented by atomic oxygen, a strong erosive agent. This is known to induce oxidative decomposition in polymeric materials, resulting in erosion phenomena as well as morphological and chemical alterations. Carbon-based nanomaterials are already intensively used in spacecraft manufacturing as structural materials (brake disks, vehicle nose tips, etc.) [23], but could be exploited for corrosion mitigation in space thanks to their peculiar features [24]. A comprehensive discussion of the pathways responsible for material degradation in the LEO environment is reported in the review by Weerasinghe et al. [25], which also focuses on the findings from state-of-the-art in-flight and simulated experiments involving carbon nanocomposites for aerospace technology.

In addition to carbon nanocomposites, as reported by Bram et al. [26], the durability of polymers under harsh LEO conditions could also be improved by incorporating polyhedral oligomeric silsesquioxane (POSS). In particular, the study focused on epoxy resins as polymeric matrix and the role of POSS in improving their resistance through its self-passivation under atomic oxygen bombardment, forming a SiO_2_ layer.

Long-term space projects and missions will also include the presence of humans for both deep space explorations (crew and scientists) and commercial spaceflights (passengers). In this context, the development of nanomaterials must also deal with the crew and passengers’ potential health issues. A trivial health issue could be the occurrence of a wound, which is easily treated on Earth but not in space. The work by Zagni et al. [27] aimed to develop a biocompatible poly-2-hydroxyethylmethacrylate system containing halloysite nanotubes as a container for Thymol, a drug that has wound-healing properties. The miniaturization of the system and its loading and releasing capabilities make it a promising skin repair system/solution for space missions. On the other hand, the extreme physiological conditions that space crews will have to face can determine stressful conditions that call for constant monitoring to prevent them from becoming critical, especially during long-term missions. Monitoring secreted hormones through wearable sensors might be a solution. With regard to this, Trusso Sfrazzetto et al. [28] discuss different smart optical and electrochemical nanosensors based on nanomaterials suitable for the detection of stress-related cortisol levels in biological samples.

In view of the future colonization of satellites and other planets, space agencies must also work on pollution reduction solutions in closed environments. As is well known on Earth, indoor pollution is emitted by both furniture and human activities, resulting in the contamination of both gas and liquid phases. In addition to the risks that arise from daily exposure to pollutants, a major concern for long-term spaceflights is also related to the recovery of water and air [29]. Indeed, current water and air remediation technologies [30,31] are hard to maintain without spare parts, so they might be supported by passive systems to prolong their lives. Photocatalytic nanosystems capable of depolluting fluids from xenobiotic waste using low-energy indoor treatments could be a potential solution not only for extraterrestrial human habitats but also for underground terrestrial infrastructures that are not directly exposed to sunlight. Exploiting advanced oxidation processes, Mezzina et al. [32] reported on the development of a filmable thermoplastic nanosystem based on copolyacrylates as the polymeric matrix and titanium dioxide as the photocatalytic moiety. To provide photodegradation efficiency also through indoor light sources, a porphyrin macromonomer was used as the photosensitizer. The proposed photosensitized nanocomposite resulted efficiently in terms of photodegradation of xenobiotic pollutants in water during batch experiments performed under visible light radiation.

In conclusion, considering all the above issues to face in space environments and the potential solutions investigated, the Special Issue titled “Nanomaterials for Potential Uses in Extraterrestrial Environments” shed light on the important role that nanomaterials play in the design of future space colonization challenges, inspiring the engineering of new nanomaterials and their applications in interdisciplinary fields.
